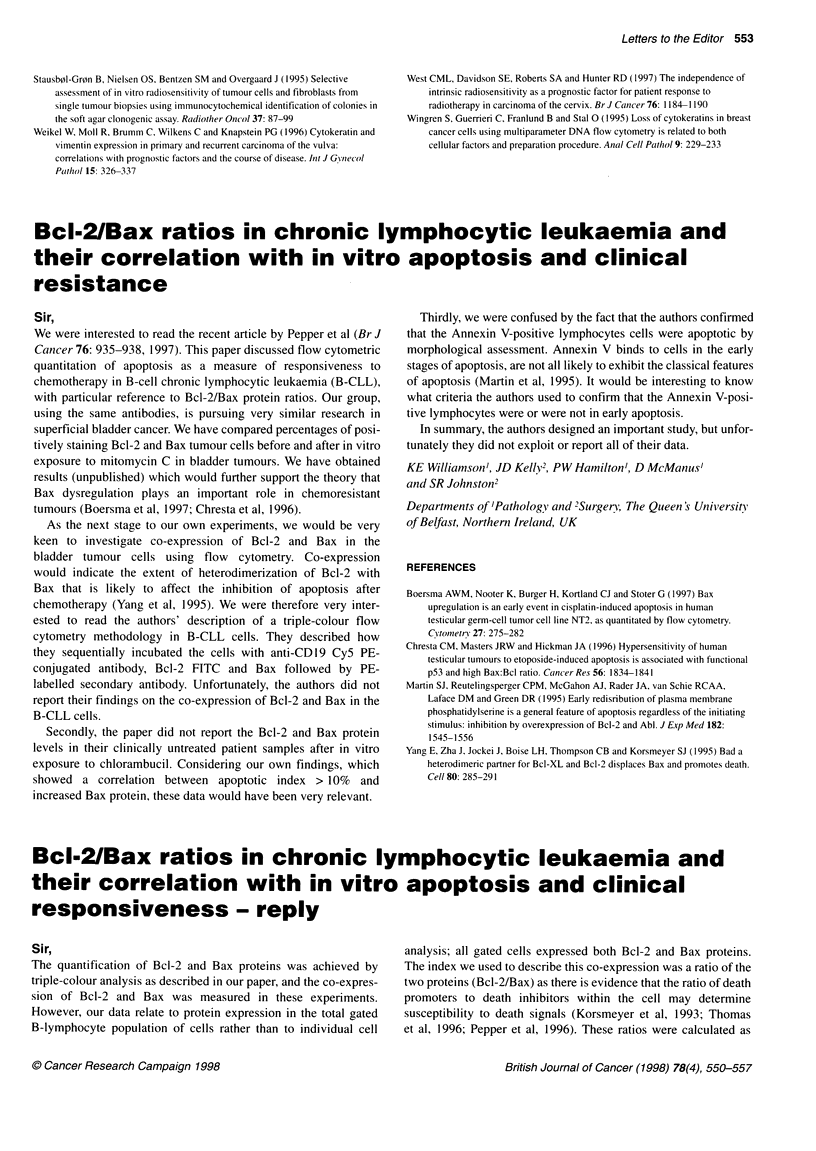# Bcl-2/Bax ratios in chronic lymphocytic leukaemia and their correlation with in vitro apoptosis and clinical resistance.

**DOI:** 10.1038/bjc.1998.533

**Published:** 1998-08

**Authors:** K. E. Williamson, J. D. Kelly, P. W. Hamilton, D. McManus, S. R. Johnston


### Bcl-2/Bax ratios in chronic lymphocytic leukaemia and their correlation with in vitro apoptosis and clinical resistance

**Sir,**

We were interested to read the recent article by Pepper et al (*Br J Cancer* **76**: 935–938, 1997). This paper discussed flow cytometric quantitation of apoptosis as a measure of responsiveness to chemotherapy in B-cell chronic lymphocytic leukaemia (B-CLL), with particular reference to Bcl-2/Bax protein ratios. Our group, using the same antibodies, is pursuing very similar research in superficial bladder cancer. We have compared percentages of positively staining Bcl-2 and Bax tumour cells before and after in vitro exposure to mitomycin C in bladder tumours. We have obtained results (unpublished) which would further support the theory that Bax dysregulation plays an important role in chemoresistant tumours (Boersma et al, 1997; Chresta et al, 1996).

As the next stage to our own experiments, we would be very keen to investigate co-expression of Bcl-2 and Bax in the bladder tumour cells using flow cytometry. Co-expression would indicate the extent of heterodimerization of Bcl-2 with Bax that is likely to affect the inhibition of apoptosis after chemotherapy (Yang et al, 1995). We were therefore very interested to read the authors' description of a triple-colour flow cytometry methodology in B-CLL cells. They described how they sequentially incubated the cells with anti-CD19 Cy5 PE-conjugated antibody, Bcl-2 FITC and Bax followed by PE-labelled secondary antibody. Unfortunately, the authors did not report their findings on the co-expression of Bcl-2 and Bax in the B-CLL cells.

Secondly, the paper did not report the Bcl-2 and Bax protein levels in their clinically untreated patient samples after in vitro exposure to chlorambucil. Considering our own findings, which showed a correlation between apoptotic index > 10% and increased Bax protein, these data would have been very relevant.

Thirdly, we were confused by the fact that the authors confirmed that the Annexin V-positive lymphocytes cells were apoptotic by morphological assessment. Annexin V binds to cells in the early stages of apoptosis, are not all likely to exhibit the classical features of apoptosis (Martin et al, 1995). It would be interesting to know what criteria the authors used to confirm that the Annexin V-positive lymphocytes were or were not in early apoptosis.

In summary, the authors designed an important study, but unfortunately they did not exploit or report all of their data.

*KE Williamson[1], JD Kelly[2], PW Hamilton[1], D McManus[1] and SR Johnston[2]*

*Departments of [1]Pathology and [2]Surgery, The Queen's University of Belfast, Northern Ireland, UK*

**REFERENCES**

Boersma AWM, Nooter K, Burger H, Kortland CJ and Stoter G (1997) Bax upregulation is an early event in cisplatin-induced apoptosis in human testicular germ-cell tumor cell line NT2, as quantitated by flow cytometry. *Cytometry* **27**: 275–282

Chresta CM, Masters JRW and Hickman JA (1996) Hypersensitivity of human testicular tumours to etoposide-induced apoptosis is associated with functional p53 and high Bax:Bcl ratio. *Cancer Res* **56**: 1834–1841

Martin SJ, Reutelingsperger CPM, McGahon AJ, Rader JA, van Schie RCAA, Laface DM and Green DR (1995) Early redisribution of plasma membrane phosphatidylserine is a general feature of apoptosis regardless of the initiating stimulus: inhibition by overexpression of Bcl-2 and Abl. *J Exp Med* **182**: 1545–1556

Yang E, Zha J, Jockei J, Boise LH, Thompson CB and Korsmeyer SJ (1995) Bad a heterodimeric partner for Bcl-XL and Bcl-2 displaces Bax and promotes death. *Cell* **80**: 285–291